# Protective effect of glucagon-like peptide-1 mediated by ultrasound microbubbles on myocardial injury in rats with diabetic cardiomyopathy

**DOI:** 10.1080/21655979.2021.2022270

**Published:** 2022-01-22

**Authors:** Yanjie Liu, Li Chen, Hao Wu, Hebin Zhang

**Affiliations:** a Department of Ultrasound, The Second Affiliated Hospital of Zhejiang Chinese Medical University, Hangzhou, China; b Department of Ultrasound, The Affiliated Hospital of Hangzhou Normal University, Hangzhou, China

**Keywords:** Glucagon-like peptide-1 receptor agonist, ultrasound microbubble, diabetic cardiomyopathy, combination therapy, rat

## Abstract

Current study was performed to investigate the therapeutic effects of UTMD combined with Semaglutide, a GLP-1 R agonist, on DCM in GK rats. After 6-week intervention, all rats were examined by conventional echocardiography, and the indicators of papillary muscle horizontal wall were measured. At the end of experiment, the DCM rats were sacrificed, and then blood samples were taken to detect blood lipids and the activities of oxidative stress-related factors in myocardial tissues. Moreover, the myocardial tissues were also taken to observe histomorphological changes and myocardial fibrosis via H&E and Masson staining, respectively. Furthermore, the mRNA and protein levels of PPAR-γ and NF-κB in myocardial tissues were detected. As a result, the glycometabolism and the cardiac functions of DCM rats received combination therapy of GLP-1 R agonist and UTMD were improved. Significantly increased SOD and GSH-Px as well as significantly decreased MDA levels were also observed in rats of combined group compared with others. In addition, both mRNA and protein levels of PPAR-γ and NF-κB were significantly lower than those of the model control ones and Semaglutide microbubbles alone (all *P* < 0.001). Further pathological histology analysis demonstrated that combination therapy effectively ameliorated fibrosis and myocardial morphological changes of DCM rats. In summary, UTMD combined with Semaglutide can effectively protect cardiac function in rats with DCM by significantly alleviating myocardial fibrosis and oxidative stress.

**Abbreviations:**

Ultrasound-targeted microbubble destruction, UTMD; glucagon-like peptide-1 receptor, GLP-1R; diabetic cardiomyopathy, DCM; Goto-Kakizaki, GK; velocity vector imaging, VVI; left ventricular end-diastolic diameter LVIDd; left ventricular end-systolic diameter, LvIDs; left ventricular end-diastolic pressure, LVEDP; fractional shortening, LVFs; left ventricular ejection fraction, LVEF; mean peak radial velocity, Vs; radial strain, Sr; radial strain rate, SRr; superoxide dismutase, SOD; malondialdehyde, MDA; glutathione peroxidase, GSH-Px; peroxisome proliferator-activated receptor-γ, PPAR-γ; nuclear transcription factor κB, NF-κB; insulin resistance, IR; total cholesterol, TC; total triglycerides, TG; creatine kinase, CK; lactate dehydrogenase, LDH; cardiac troponin I, cTnI; collagen volume fraction, CVF; Hematoxylin eosin, H&E.

## Introduction

It is estimated that there are more than 500 million people with diabetes worldwide in 2020, which is expected to increase to 693 million by 2045 [1,2]. Long-term diabetes is associated with many complications, of which approximately 75% of deaths in diabetic patients are caused by cardiovascular diseases [3,4]. Patients with diabetes have increased cardiovascular morbidity and mortality compared with non-diabetic patients [5]. This increased risk of cardiovascular disease is due to microvascular and macrovascular complications resulting from diabetes, often leading more rapidly to more severe and widespread atherosclerotic disease and consequently to further heart failure when accompanied by other cardiovascular risk factors such as hypertension, dyslipidemia, activation of neurohormonal and inflammatory mechanisms [6,7]. The clinical definition of a disease process in which this diabetes directly affects the myocardium has added a definition called ‘diabetic cardiomyopathy (DCM)’ [6]. DCM is defined as diastolic and systolic dysfunction independent of coronary heart disease, hypertension, and other cardiac diseases [8]. The etiology of DCM includes two major defects in diabetes, namely insulin resistance (IR) and hyperglycemia causing a spectrum of specific cardiomyocyte abnormalities [9]. IR and hyperglycemia are thought to trigger a series of compensatory and decompensated responses that ultimately lead to impairment of cardiac function [9]. Complex pathophysiological changes ultimately lead to cardiac structural changes in DCM, acting together on cardiac remodeling [10]. Patients with DCM have the following changes in cardiac structure: stasis interstitium and perivascular fibrosis. Interstitial fibrosis is a histological and early feature of DCM and leads to reduced cardiac compliance [6,11]. Interstitial fibrosis is an alternative process to focal myocardial cell necrosis and a result of inflammatory connective tissue cells responding to pathological triggers [12].

Recent studies reported that the GLP-1 could exert improved effects on the angiocarpy functions, which mainly due to that the GLP-1 R agonist directly stimulates cardiomyocytes-related signaling pathways via the GLP-1 R distributed in cardiac cells [13,14]. Moreover, GLP-1 R agonists may also indirectly improve the function of cardiomyocytes by controlling metabolic factors, including GIP, glucagon, insulin, glucose levels, or by free fatty acid levels [15]. In addition, GLP-1 R agonists can also stimulate CNS regions that play an important role in controlling cardiovascular efficiency and help to improve or restore the functions [16]. As a new type of transport carrier, microbubble contrast agent can carry drugs, cytokines, genes and other substances, burst microbubbles under certain energy ultrasound irradiation, and accurately release transport substances in target tissues, so as to achieve the purpose of targeted therapy [17,18].

Over the last decade or so, there have been multiple GLP-1 R agonists, with Semaglutide providing the best cardiovascular benefits in patients with diabetes [19]. Given the safety benefits of GLP-1 R agonists, we hypothesized microbubbles containing Semaglutide, which burst under ultrasound irradiation, could increase drug concentration around myocardial tissue and protect myocardial tissue more effectively. Therefore, the purpose of this study was to investigate the protective effect of ultrasound combined with microbubbles on myocardial injury in rats with diabetic cardiomyopathy, and its mechanism of regulating cardiac function and injury, providing new ideas for the treatment of DCM.

## Methods and materials

### Materials and animals

SPF grade GK rats (male, ~300 g, 8–10 weeks old) were purchased from the Laboratory Animal Center of Zhejiang Chinese Medical University. All animal experimental experiments were performed following the guidelines for Animal Care and Use Committee. Microbubble contrast agents were purchased from Braco (Italy). Creatine kinase, lactate dehydrogenase, and cardiac troponin I assay kits were purchased from Nanjing Jiancheng Bioengineering Institute (China). CXCR4 rabbit anti-mouse monoclonal antibody was purchased from Abcam (USA). Trizol RNA extraction reagent, Real-timeScript™ reverse transcription kit, and SYBR oride was purchased from Sigma-Aldrich (USA). SonoVue injection kit was purchased from BRACCO INTERNATIONAL BV. (Spain).

### Preparation of GLP-1 R agonist microbubbles

According to the instructions for use of the kit, 5 mL of injection-grade normal saline (0.9% NaCl) containing 100 μg Semaglutide was added to the SonoVue vial, shaken vigorously for 20 s until the lyophilized powder was completely dispersed, and allowed to stand at room temperature for 115 min for incubation, during which several gentle oscillations were performed to obtain ultrasound microbubbles co-loaded with Semaglutide (SonaVue-semaglutide).

### Animal experiment

The rats were intraperitoneally injected with adriamycin at the dose of 2.5 mg/kg, and the model control group and the healthy control group were injected with an equal volume of normal saline (1 mg/mL) once a week for 6 weeks. The dose of adriamycin was appropriately adjusted according to the weight change of the animals. After successful modeling, GLP-1 R agonist and GLP-1 R agonist microbubbles + UTMD groups were administered via s.c. and tail i.v. injection at the dose of 0.2 mg/kg, and the UTMD alone treatment group was given an equal volume of saline. Then the rats in the UTMD and Semaglutide microbubbles + UTMD groups were anesthetized by intraperitoneal injection of 10% chloral hydrate, the precordium was shaved. Then the probe was further placed in the precordium of rats, and a short-axis view was taken at the papillary muscle level with a focal depth of 3.5–4.0 cm. When a large number of microbubbles were seen in the myocardium, the microbubbles were repeatedly burst with the MBD function of the machine (mechanical index MI = 1.9) until the microbubbles completely disappeared. The intervention was performed on the 4th and 6th day after weekly injection for 6 weeks. Fasting blood glucose and body weight were measured at fixed times before and weekly after the intervention and recorded. Changes in glycosylated hemoglobin and glucose tolerance were performed using biochemical analyzer and classical oral glucose tolerance test methods, respectively. The contents of TC and TG in serum were determined by ELISA methods.

### Evaluation of myocardial biochemistry and cardiac functions

Serum samples were taken before intervention, 2, 4 and 6 weeks later, and CK, LDH and cTnI contents in serum were measured by ELISA according to the instructions of kits. After 6 weeks of intervention, rats in each group were anesthetized by intraperitoneal injection of 1 mL/kg of 2% pentobarbital sodium. LVIDd, LVEDP and LVEF were measured by M-mode ultrasonography. At the end of cardiac function assessment, all the body weights of experimental rats were weighed and recorded. The rats in each group were sacrificed, the thoracic cavity of the rats was opened, the ascending aorta was clamped, and the heart was rapidly taken to remove the great vessels of the rat heart. Then the residual blood in the cardiac chamber was removed by flushing with saline, the water was removed with filter paper. The rats were further placed on a microbalance for weighing, and the heart/body weight ratio (HW/BW) was calculated. After weighing the heart of rats, right ventricle was cut off, the left ventricle was preserved, and part of the myocardium was harvested perpendicular to the midpoint of the long axis symmetry of the left ventricle of the heart and placed in 4 paraformaldehyde for fixation, embedded in paraffin 24 h later, and sectioned continuously for 5 mm. H&E staining and Masson staining were performed according previously reported methods [20], and placed under a high-resolution light microscope to observe the morphological changes of myocardial tissues. The degree of myocardial fibrosis, and the CVF was calculated via using the ImageProPlus 6.0 software.

After freezing and grinding of myocardial tissues, half were added with lysate to extract protein, followed by Western blotting to detect the protein expression of PPARγ and NF-κB in myocardial tissues. Subsequently, the protein samples were separated by 1% SDS-PAGE electrophoresis, then transferred to PVDF membranes at 300 mA constant current, blocked with 5% skimmed milk powder for 1 h at room temperature, and then incubated with human anti-CXCR4 monoclonal antibody overnight at 4°C. The next day, PVDF were washed with TBST three times, 5 min/time, incubated with human horseradish enzyme-labeled secondary antibody for 1 h at room temperature, and then washed with TBST three times, 5 min/time, and the signal was detected by ECL chemiluminescence agent luminescence. The other half of the tissue was used to determine mRNA. The 1 mL of TRIozl reagent was added, repeatedly blown and aspirated and transferred into an EP tube, total RNA was extracted from the tissue according to the instructions for use of the TRIzol kit, and RNA quality and concentration were measured by spectrophotometer. Reverse transcription and real-time PCR assays were performed following the TaKaRa reagent instructions. β-actin, reverse primer: 5ʹ-CATCCGTAAAGACCTCTATGCCAAC-3ʹ, Forward primer: 5ʹ-ATGGAGCCACCGATCCACA-3ʹ. PPAR-γ, reverse primer: 5ʹ-GGAGCCTAAGTTTGAGTTTGCTGTG-3ʹ, Forward primer: 5ʹTGCAGCAGGTTCTCTTGGATG 3ʹ. NF-κB, reverse primer: 5ʹ-GGATGGCTACTATGAGGCTGAC-3ʹ, Forward primer: 5ʹ-CTAATGGCTTGCTCCAGGTCTC-3ʹ.

### Statistical methods

SPSS 16.0 software was used for statistical analysis of the data of each group. Sample size estimation was performed by G*Power (3.1.2) with an alpha level of 0.05 and power of 0.8. The measurement data were expressed as Mean± S.E.M. The two independent samples f test was used for comparison. P < 0.05 was considered statistically significant.

## Results

In this study, we hypothesized microbubbles containing Semaglutide, which burst under ultrasound irradiation, could increase drug concentration around myocardial tissue and protect myocardial tissue more effectively. To prove this hypothesis, we investigate the protective effect of ultrasound combined with microbubbles on myocardial injury in rats with diabetic cardiomyopathy, and its mechanism of regulating cardiac function and injury, providing new ideas for the treatment of DCM.

### Effects of combined GLP-1 R agonist with UTMD on general indexes of the DCM rats

**Figure 1.**
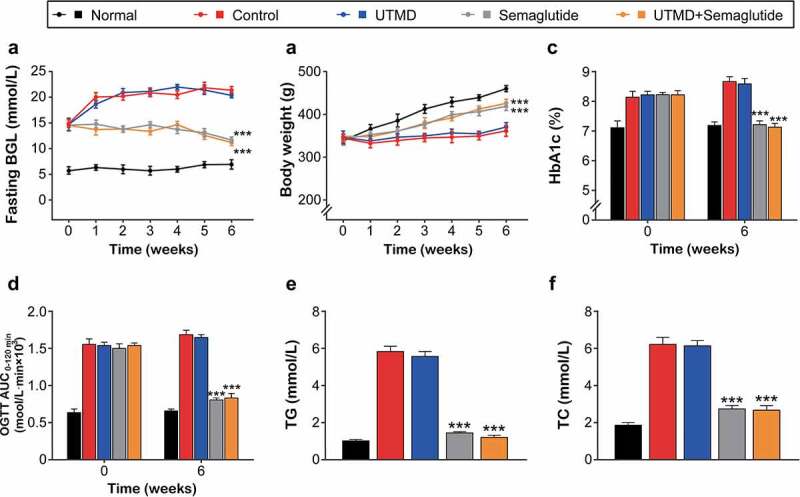
Effects of combined GLP-1 R agonist with UTMD on general indexes of the diabetic rats. The (a) fasting BGL; (b) body weight; (c) HbA1c; (d) OGTT AUC; (e) TG and (f) TC levels of the model rats. All data are expressed as means with error bars as standard deviations (n = 8). *** denotes P < 0.001 vs. Model control group.

Firstly, the effects of combination therapy on diabetes-related parameters in GK rats were examined. Fasting blood glucose levels continued to increase in the model groups received the saline or UTMD treatment during the 6-week intervention period, while blood glucose was effectively controlled in the group treated with combined UTMD with Semaglutide, but there was no significant advantage over that of the Semaglutide alone treated group (Figure 1a). After 6 weeks of intervention, diabetic rats in model control group and UTMD group had significantly increased body weight compared to the Semaglutide or UTMD+Semaglutide group, while there was no significant difference between two Semaglutide intervention groups (Figure 1b). The improvement on %HbA1c, glucose tolerance and blood lipid profiles in GK rats were all similar to the fasting BGLs, and the ones received the combination treatment were significantly improved compared with the control group, but not significant higher compared with those of the Semaglutide alone group (Figure 1c-f).

**Figure 2.**
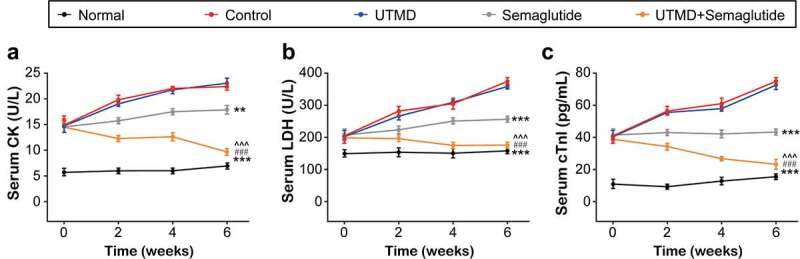
Effects of combined GLP-1 R agonist with UTMD on serum cardiac indexes of diabetic rats. The serum (a) CK; (b) LDH; (c) cTnl levels of the model rats. All data are expressed as means with error bars as standard deviations (n = 8). *, ** or *** denotes P < 0.05, 0.01 or 0.001 vs. Control group; #, ## or ### denote P < 0.05, 0.01 or 0.001 vs. Semaglutide alone group; ^, ^^ or ^^^ denote P < 0.05, 0.01 or 0.001 vs. UTMD alone group.

The serum levels of myocardial injury-related parameters were further detected and the results were showed in Figure 2. All three indicators of the saline-treated GK rats with DCM were significantly increased compared with those of the normal ones, while the combination group significantly reversed the increase of these indicators, and the improvement effect was significant compared with the two monotherapies, suggesting that the combination therapy can effectively reduce the increase of related indicators by improving myocardial injury.

### Effects of combined GLP-1 R agonist with UTMD on cardiac functions of the DCM rats

As shown in Table 1, the results of conventional ultrasound showed that LVIDs and LvIDd in DCM model group were significantly higher than those in normal control group, while LVEF and LVFS were significantly lower (all *P* < 0.05). After 6 weeks of intervention, LVEF and LVFS in combined Semaglutide with UTMD group were significantly higher than those in saline-treated DCM model group (both *P* < 0.01), while the LVIDs and LvIDd were significantly lower (*P* < 0.01 and *P* < 0.05, respectively). Compared with Semaglutide alone group, LVEF and LVFS in Semaglutide + UTMD group were significantly increased (both *P* < 0.05). As the results of VVI in Table 2, Vs, Sr and SRr in DCM model group were all significantly lower than those in normal control group. After 6 weeks of intervention, Vs, Sr and SRr in Semaglutide + UTMD group were significantly higher than those in DM model group. Moreover, the Sr and SRr in Semaglutide + UTMD group were significantly increased compared with Semaglutide alone group.

**Table 1.** Effects of combined GLP-1 R agonist with UTMD on routine ultrasound cardiac functions of the model rats

Groups	LVIDs (mm)	LVIDd (mm)	LVEF (%)	LVFS (%)
Normal	3.96 ± 0.29	7.18 ± 0.34	82.77 ± 5.16	47.22 ± 3.58
Control	5.23 ± 0.24	7.84 ± 0.21	58.52 ± 3.72	27.60 ± 3.09
UTMD	5.48 ± 0.36	7.63 ± 0.69	57.42 ± 4.87	27.81 ± 2.68
Semaglutide	4.75 ± 0.21 *	7.58 ± 0.42	69.06 ± 3.50 *	32.08 ± 3.16 ^#^
UTMD+ Semaglutide	4.05 ± 0.19 **^, #,^ ^	7.07 ± 0.31*	80.68 ± 5.36 ***^, #,^ ^^^	42.58 ± 3.24**^, #,^ ^

All data are expressed as means with error bars as standard deviations (n = 8). *, ** or *** denotes P < 0.05, 0.01 or 0.001 vs. Control group; #, ## or ### denote P < 0.05, 0.01 or 0.001 vs. Semgalutide alone group; ^, ^^ or ^^^ denote P < 0.05, 0.01 or 0.001 vs. UTMD alone group.

**Table 2.** Effects of combined GLP-1 R agonist with UTMD on VVI indicators of model rats. All data are expressed as means with error bars as standard deviations (n = 8)

Groups	Vs (cm/s)	Sr (%)	SRr (s^−1^)
Normal	1.12 ± 0.27	29.05 ± 2.34	3.27 ± 0.66
Control	0.44 ± 0.09	8.04 ± 1.35	1.42 ± 0.23
UTMD	0.48 ± 0.13	8.29 ± 2.51	1.53 ± 0.53 ^#^
Semaglutide	0.69 ± 0.16 *	14.69 ± 1.24**	1.82 ± 0.39 ^#^
UTMD+Semaglutide	0.96 ± 0.21 ***^, ##,^ ^^^	22.54 ± 4.31***^, ###,^ ^^^	2.92 ± 0.63 **^, #,^ ^

*, ** or *** denotes *P* < 0.05, 0.01 or 0.001 vs. Control group; #, ## or ### denote *P* < 0.05, 0.01 or 0.001 vs. Semgalutide alone group; ^, ^^ or ^^^ denote *P* < 0.05, 0.01 or 0.001 vs. UTMD alone group.

### Effects of combined GLP-1 R agonist with UTMD on heart tissues of the DCM rats

Compared with the normal control group, the body weight of the other four DCM model groups were all significantly decreased (all *P* < 0.01, Table 3). Interestingly, the decreased body weight of the Semaglutide + UTMD group was reversed compared to that of the saline-treated DCM model group, but the difference was not statistically significant (*P* > 0.05). Moreover, the heart mass of the combination group was significantly reduced (*P* < 0.05) compared with the normal control group, while that of the other groups did not exhibited statistically significant difference (all *P* > 0.05). Furthermore, the Hw/Bw of the DCM model group, Semaglutide group and UTMD group were all significantly higher than that of the normal control group (all *P* < 0.05), and the Hw/Bw of the Semaglutide + UTMD group was significantly decreased compared with the other DCM model groups (all *P* < 0.05).

**Table 3.** Effects of combined GLP-1 R agonist with UTMD on the body and heart weights. All data are expressed as means with error bars as standard deviations (n = 8)

Groups	BW (g)	HW (mg)	HW/BW (mg/g)
Normal	468.56 ± 15.95	1042.33 ± 59.09	2.23 ± 0.15
Control	339.29 ± 43.45	1205.32 ± 174.26	3.55 ± 0.35
UTMD	345.86 ± 29.35	1221.26 ± 124.93	3.53 ± 0.26
Semaglutide	419.08 ± 22.95 ***	991.99 ± 106.24***	2.36 ± 0.27 **
UTMD+ Semaglutide	428.46 ± 20.72 ***^,^ ^^^	971.71 ± 94.08***^,^ ^^^	2.26 ± 0.23 **^,^ ^^

*, ** or *** denotes P < 0.05, 0.01 or 0.001 vs. Control group; ^, ^^ or ^^^ denote P < 0.05, 0.01 or 0.001 vs. UTMD alone group.

**Figure 3.**
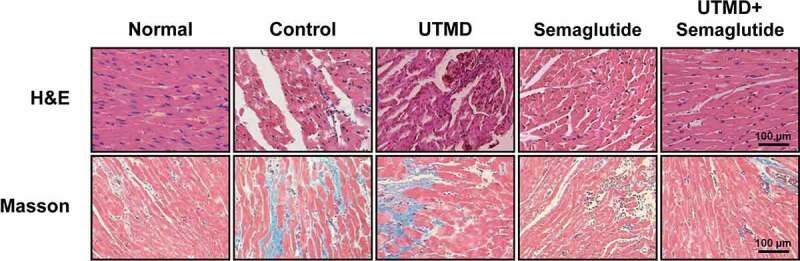
Effects of combined GLP-1 R agonist with UTMD on the tissue structure and fibrosis. (a) H&E and (b) Masson staining analysis of heart tissues from the rats in different groups.

As shown in Figure 3, H&E staining of left ventricular myocardial tissue showed that the myocardial fibers of healthy rats were arranged neatly and the cells were arranged evenly. While in the DCM model group, the myocardial tissue was clearly damaged, some myofibrils were dissolved, and the myocardial fibers were broken, cell space was significantly widened. On the contrary, the intercellular space was widened and a few myocardial fibers were broken; the myocardial fibers were relatively neatly arranged in the Semaglutide combined with UTMD group. There was no destruction of myocardial fibers with the normal intercellular spaces and without edema or vacuolization.

Masson staining analysis demonstrated that, in the normal group, the myocardial cells were neatly arranged, the nucleus was dark blue, the collagen fibers were bright green, and only a small amount of distribution was observed in the myocardial interstitium and around the blood vessels. In the model group, the myocardial cells were hypertrophic, necrotic, and disorganized, the bright green collagen fibers in the myocardial interstitium were significantly increased, and there was significant myocardial fibrosis. The disarrangement of myocardial cells in combination treatment group was significantly improved, the bright green collagen fibers in myocardial interstitium were also significantly reduced compared with the model group, and the improvement effect was significantly better than that of the two monotherapy groups.

### Effects of combined GLP-1 R agonist with UTMD on oxidative stress in the DCM rats

**Figure 4.**
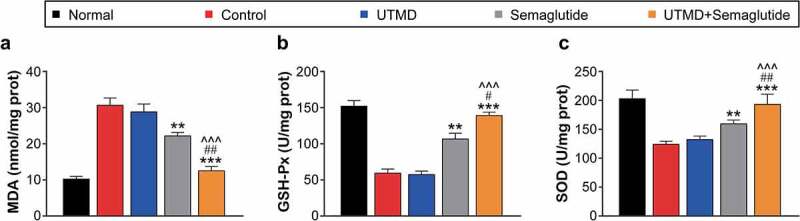
Effects of combined GLP-1 R agonist with UTMD on oxidative stress in model rats. The levels (a) MDA, (b) GSH-Px and (c) SOD were measured. All data are expressed as means with error bars as standard deviations (n = 8). *, ** or *** denotes P < 0.05, 0.01 or 0.001 vs. Control group; #, ## or ### denote P < 0.05, 0.01 or 0.001 vs. Semaglutide alone group; ^, ^^ or ^^^ denote P < 0.05, 0.01 or 0.001 vs. UTMD alone group.

As shown in Figure 4, both SOD and GSH-PX in the cardiac tissues from DCM model group were significantly lower than those in the normal rats, while the MDA level was significantly increased, suggesting that there was significant oxidative stress in the model rats. The levels of SOD and GSH-PX in rats treated with UTMD combined with Semaglutide were significantly higher than those in the model control group, close to those of the healthy ones, and the change trend of MDA was similar, suggesting that the treatment of UTMD combined with Semaglutide can effectively improve the oxidative stress response accompanied by myocardial injury.

### Effects of combined GLP-1 R agonist with UTMD on the mRNA and protein expression of PPAR-γ and NF-κB in cardiac tissues

**Figure 5.**
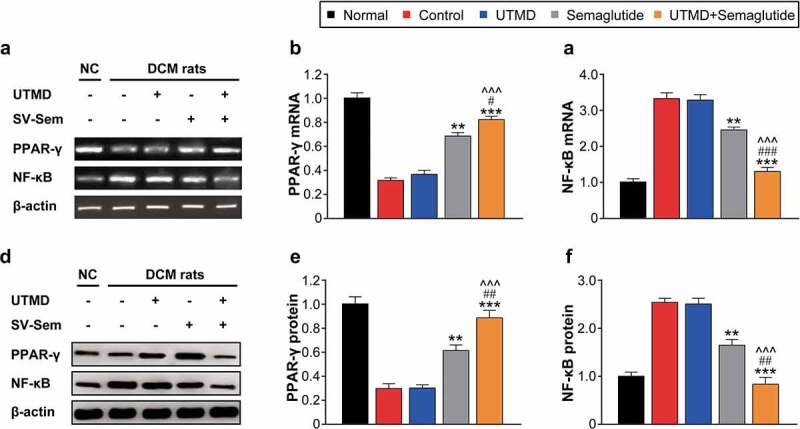
Effects of combined GLP-1 R agonist with UTMD on the expression of mRNA and protein of PPAR-γ and NF-κB. The (a) agarose gel image and mRNA expression of (b) PPAR-γ and (c) NF-κB; The (d) protein gel image and protein expression of (e) PPAR-γ and (f) NF-κB. All data are expressed as means with error bars as standard deviations (n = 3). *, ** or *** denotes P < 0.05, 0.01 or 0.001 vs. Control group; #, ## or ### denote P < 0.05, 0.01 or 0.001 vs. Semaglutide alone group; ^, ^^ or ^^^ denote P < 0.05, 0.01 or 0.001 vs. UTMD alone group.

The mRNA and protein expression levels of PPAR-γ and NF-κB in the myocardial tissues of rats in each group were further determined, and the results are shown in Figure 5. Compared with the normal control group, the expression levels of PPAR-γ mRNA and protein in each model were generally lower, with the least decrease in the combination, and were significantly better than those in the two monotherapy model groups. Similarly, the combined treatment effectively reversed the significantly increased mRNA and protein levels of NF-κB, and the improvement was significantly better than other groups, and close to the normal control group. The above results collectively suggested that combination therapy can reduce hyperglycemia-induced myocardial fibrosis by increasing the expression of PPAR-γ and inhibiting the activity of NF-κB in myocardial tissues of DCM rats, thereby controlling inflammatory response.

## Discussion

DCM is a serious complication of diabetes, and non-myocardial cell remodeling occurs in its pathogenesis, resulting in cardiac insufficiency and decreased pumping ability, triggering complications such as heart failure and shock [8,9]. So far, the exact mechanism of the pathogenesis of DCM has not been fully elucidated and is thought to be the result of a combination of factors including hyperglycemia, hyperlipidemia, and inflammation [21]. It has been reported in the literature that the increase of oxidative stress is one of the main factors in the pathogenesis of DCM, and oxidative stress is in turn an important cause of myocardial fibrosis [22,23]. Although there is a series of clinical treatment measures [6]. However, it is generally difficult for DCM patients to obtain satisfactory therapeutic results [6]. Therefore, how to effectively prevent and treat DCM has become a hot spot in clinical research, and it is of great clinical significance to actively study and develop new drugs and methods that can effectively prevent and treat DCM.

As a new type of transport carrier, microbubble contrast agent can carry drugs, cytokines, genes and other substances, burst microbubbles under certain energy ultrasound irradiation, and accurately release transport substances in target tissues, so as to achieve the purpose of targeted therapy [17]. Before the current research start, low-frequency ultrasound was used for the experiment of in vivo intervention in rats according to the reference [24], and the results showed that there was no significant difference in CK, LDH and cTnI levels between rats after continuous intervention twice a day for 6 days (data not shown), indicating that continuous intervention for 12 times in a short time with strong ultrasound irradiation at 1 W/Cm2 was noninvasive and safe for rat myocardial tissues.

Combined with some previous publications, we believe that GLP-1 R agonists have the potential to be a high potential therapeutic option for treating DCM [25,26]. However, considering that the conventional administration method of GLP-1 R agonist is subcutaneous administration, which is difficult to enrich to the heart. Previous preliminary experiment for investigating the effects of s.c injection of Semaglutide on the DCM demonstrated the therapeutic potency of Semaglutide (data not shown). Therefore, we proposed that it is possible to achieve effective targeted therapy by combining microbubble technology for site-directed therapy, but it still needs to be evaluated by *in vivo* experiments. In this study, we hope to investigate the potential preventive and therapeutic effects of combined GLP-1 R agonist with UTMD on diabetic cardiomyopathy in rats.

In this study, SonoVue microbubbles loaded with Semaglutide were prepared by non-covalent physical binding, and ultrasound energy was used to target and rupture SonoVue microbubbles loaded with Semaglutide in myocardial tissue, so as to enrich Semaglutide into myocardial tissue and exert its preventive and therapeutic effects on DCM. Firstly, the fasting blood glucose, glycosylated hemoglobin, blood lipids and other indicators of DCM rats were all significantly increased compared with healthy ones, while after 6 weeks of intervention with combination therapy, these symptoms were significantly improved compared with the model control group, but there was no significant advantage compared with the Semaglutide alone group. After 6 weeks of intervention, LVEF, LVFS, Vs, Sr and SRr in combination group were significantly higher than those in DCM model group (all P < 0.05), while LVIDs and LVIDd were significantly smaller (P < 0.01 or P < 0.05, respectively). Compared with Semaglutide alone group, LVEF, LVFS, Sr and SRr in combined treatment group were significantly higher (all *P* < 0.05). Results of H&E and Masson staining showed that the myocardial tissues of DCM model group were severely damaged, the myocardial interstitial collagen fibers were significantly increased and disorganized, the myocardial cell interspace was significantly widened, and vacuolization was observed. Moreover, the Hw/Bw ratio of DCM rats was significantly higher than of healthy ones (*P* < 0.01). Our study confirmed that long-term high glucose state can cause significant myocardial fibrosis, myocardial fibrosis leads to increased ventricular wall stiffness, decreased compliance, and significantly enlarged ventricular chamber, which significantly decreases cardiac function LVEF, LVFS, Sr, and SRr values. Significantly, combination therapy effectively reverses above mentioned symptoms and exhibited better improvement compared with two monotherapies. Ultrasound microbubbles as contrast agents allow ultrasound visualization of myocardial tissue, and the cavitation and mechanical effects produced by ultrasound microbubble burst allow temporary opening of small pores in the local endothelial cell membranes and capillaries of the myocardium in response to diagnostic or therapeutic ultrasound, through which Semaglutide diffuses into the myocardial tissue, thereby targeting GLP-1 R agonist to the myocardium and exerting its biological function via GLP-1 R.

SOD is an antioxidant metalloenzyme existing in organisms, which can catalyze the dismutation of superoxide anion radicals to generate oxygen and hydrogen peroxide [27]; GSH-Px is an important peroxidolytic enzyme that can protect the structure and function of cell membranes from peroxide interference and damage, both of which play a crucial role in the balance of oxidation and anti-oxidation in the body [28]. MDA content is an important parameter reflecting the potential antioxidant capacity of the body, which can reflect the rate and intensity of peroxidation, and can also indirectly reflect the degree of tissue peroxidation damage [29]. The activities of SOD and GSH-PX in myocardial tissue were further examined, and the results exhibited significantly decrease in the myocardial tissues of DCM model rats (both P < 0.01), while the MDA content was significantly increased (P < 0.01). After combined treatment intervention, the activities of SOD and GSH-PX increased and the MDA content decreased in the myocardial tissues, and there were statistically significant differences relative to the saline-treated model ones (all *P* < 0.05). Moreover, the combination therapy holds significant efficacy advantages over monotherapy on multiple parameters, suggesting that SonoVue microbubbles loaded with Semaglutide at the targeted rupture of myocardial tissue using ultrasound energy can effectively enrich Semaglutide to myocardial tissue and play a role in protecting cardiac tissues from damage.

Our results suggest that Semaglutide combined with ultrasound microbubbles may have great therapeutic potential for the treatment of diabetic cardiomyopathy and encourage further translational efforts to ultimately benefit diabetic patients. However, additional pre-clinical studies (including in larger animals) are required to validate the efficiency and safety of Semaglutide microbubbles in the diabetic setting, and investigate the optimal dose and route of administration to be used. Finally, as Semaglutide is exogenous, a better understanding of the effects of Semaglutide-loaded microvesicle transfer on non-cardiac tissues and organs needs to be clarified. In addition, the potential side effects common to all other pro-angiogenic and pro-survival factors, such as tumor and ocular angiogenesis and arthritic angiogenesis, should also be considered.

## Conclusion

Current study demonstrated that the combination of ultrasound microbubbles loaded with Semaglutide and UTMD can effectively treat diabetic myocardial injury and protect the cardiac functions of DCM rats, and the mechanism may be related to the improvement of myocardial antioxidant capacity and slow the formation of myocardial fibrosis by the Semaglutide enriched in the heart. Current combination therapy is expected to be a new method for clinical treatment of DCM.

## Highlight

- Semaglutide combined UTMD significantly improved cardiac functions in DCM rats;
- Combined therapy effectively ameliorated morphological changes in myocardium of DCM rats;
- Combined therapy effectively improved the oxidative stress response in DCM rats;
- Combined therapy reduced myocardial fibrosis by affecting PPAR-γ/NF-κB expression in DCM rats
